# Anxiety and quality‐of‐life for parents of children with undiagnosed rare conditions: A multi‐site quantitative survey study

**DOI:** 10.1002/jgc4.70085

**Published:** 2025-08-06

**Authors:** Ria Patel, Bettina Friedrich, Saskia C. Sanderson, Holly Ellard, Celine Lewis

**Affiliations:** ^1^ UCL Medical School University College London London UK; ^2^ Population, Policy and Practice Department UCL Great Ormond Street Institute of Child Health London UK; ^3^ UCL Institute of Epidemiology and Health Care London UK; ^4^ Department of Biostatistics and Health Informatics, Institute of Psychiatry, Psychology and Neuroscience King's College London London UK; ^5^ Department of Behavioural Science and Health University College London London UK; ^6^ NIHR Mental Health Translational Research Collaboration (MH‐TRC) Mental Health Mission; ^7^ NHS North Thames Genomic Laboratory Hub Great Ormond Street Hospital for Children NHS Foundation Trust London UK

**Keywords:** anxiety, diagnostic odyssey, family functioning, parent, quality‐of‐life, resilience, tolerance of uncertainty, undiagnosed condition, whole genome sequencing

## Abstract

Parenting a child with a rare undiagnosed genetic condition can impact psychological well‐being, including anxiety and health‐related quality‐of‐life. We conducted a multi‐site quantitative survey with parents to understand which parent and child characteristics are predictive of poorer psychological outcomes. 1366 surveys were sent out across seven NHS Trusts in England; 383 were returned and included in analysis (27% response rate). We used the GAD‐7 to measure parents' generalized anxiety and the PedsQL Family Impact Module (FIM) to measure self‐reported physical, emotional, social, and cognitive functioning (the health‐related quality‐of‐life [HRQOL] summary score), communication, worry, daily activities, and family relationships (the family functioning [FF] summary score). Participant characteristics included: the 6‐item Brief Resilience Scale to measure parental resilience, a bespoke single question to assess parents' tolerance for uncertainty, the EQ‐5D‐Y‐3L to measure child health‐related quality‐of‐life, two bespoke questions to assess the perceived seriousness/consequences of the child's condition, and standard characteristics questions (e.g., age, ethnicity, education, income). Overall, parental anxiety was low (mean = 5.31; SD = 5.82, range 0–21), although 21.9% had moderate (11.4%) or severe (10.5%) anxiety. A multivariable analysis indicated that higher anxiety scores were significantly associated with younger parental age (*p* = 0.010), lower education attainment (0.004), lower resilience (*p* = 0.049), and lower tolerance for uncertainty (*p* = 0.021). FIM total scores ranged from 0 to 100 (mean = 53.68, SD 20.45). Parents scored lowest on the subscale daily activities (43.68), worry (47.29), communication (51.31), and physical functioning (52.45). *Family functioning* summary scores were significantly lower for parents of children with developmental disorders compared to other conditions (*p* = 0.016). Multivariable analysis identified that lower scores (reflecting poorer outcomes) were significantly associated with lower parental resilience and lower tolerance for uncertainty (*p* < 0.001, respectively). Our findings highlight the significant psychological burden parenting a child with a rare undiagnosed condition can have on some parents and the importance of developing tailored support strategies.

**What is known about this topic**

Parenting a child with a rare and undiagnosed condition can impact overall well‐being and ability to function in daily life. Psychological outcomes including anxiety, health‐related quality‐of‐life, and family functioning have been identified to be poorer for parents of children with complex health conditions; however, the literature is sparse when it comes to children with undiagnosed conditions.

**What this paper adds to this topic**

Our study highlights that resilience and tolerance for uncertainty are key factors significantly associated with parental psychological outcomes. Strategies to enhance resilience and improve tolerance for uncertainty are needed.

## 1 BACKGROUND

Around 50–75% of rare diseases, defined as diseases that affect fewer than 1 in 2000 people, are first identified in childhood, and the majority of those will have a genetic component (European Organisation for Rare Diseases, 2005). These conditions are often multi‐systemic and complex; however, most children are able to live at home despite requiring complex medication regimens as well as, in some cases, life‐sustaining technologies such as tracheostomies, ventilators, and/or enteral feeding tubes. The move from hospital‐based to home‐based care places a burden on parents and caregivers, an experience which has been described as *intense parenting* and is characterized by the need for extra support and resources, hands‐on care, and increased responsibilities (Medrano et al., 2013). The responsibility placed on parents and caregivers (hereon is referred to as “parents”) may impact their overall well‐being and ability to function in daily life.

Anxiety may be defined as apprehension, tension, or uneasiness that stems from the anticipation of danger, which may be internal or external (Griffin, 1990). Health‐related quality‐of‐life (HRQOL) refers to an individual's perceived physical, mental, and social well‐being as it relates to their health conditions and medical care (Brooks, 1996). It encompasses disease, disability, or treatment on daily functioning, emotional well‐being, and overall quality‐of‐life (Brooks, 1996). Intense parenting can have a detrimental impact on anxiety and HRQOL, and research has highlighted that parents of undiagnosed children with rare genetic conditions experience greater distress and reduced well‐being relative to parents of children with other chronic conditions or who are typically developing (Fitzgerald & Gallagher, 2021). For example, Driscoll et al. found that rates of depressive and anxious symptoms were elevated in female and male caregivers of children with cystic fibrosis, and found that as depressive and anxious symptoms increased, caregiver quality‐of‐life (QOL) decreased (Driscoll et al., 2009).

The literature is sparser when it comes to children with undiagnosed conditions. Quantitative research has highlighted that parents of undiagnosed children have high rates of anxiety and depression, which are significantly inversely correlated with coping self‐efficacy (McConkie‐Rosell et al., 2018). Mothers have been found to have lower QOL scores than mothers of children with a diagnosis, as well as lower QOL scores than fathers (Lingen et al., 2016). Parents who feel more uncertain about their child's undiagnosed medical condition perceive themselves to have less control and optimism (Madeo et al., 2012). Qualitative research has highlighted that parents live in a world of uncertainty and complexity, which has ramifications across the extended family (Aldiss et al., 2021). They do not have time to prioritize their own well‐being and hold back their emotions to protect themselves and others (Aldiss et al., 2021). Parents can feel anxious and frightened for the future, including concern for the health of other family members (Peter et al., 2022).

Parent and child characteristics may also influence parents' psychological state. McConkie‐Rosell et al. found that parents with older children and with a longer duration of illness were better at coping and had less depression and anxiety than parents of children with a shorter duration of illness (McConkie‐Rosell et al., 2018). In a survey study conducted with parents of children with a diagnosed yet chronic disabling disorder, it was found that depression was more frequent among mothers with low educational attainment and from low socioeconomic backgrounds (Ben Thabet et al., 2013). Parents' tolerance for uncertainty was found to be an important characteristic that impacted how well‐adjusted parents felt regarding their child's condition in a qualitative study examining the parental journey of children with ultra‐rare disorders (Stafford‐Smith et al., 2025), and the importance of mental resilience for adaptation was identified in a study of families of children with 22q11.2 deletion syndromes (Caples et al., 2024).

Recent advances in genomics using next‐generation sequencing technologies including whole genome sequencing (WGS), whereby the entirety of the genome is sequenced, are improving diagnostic rates, and the diagnostic yield of WGS for previously unsolved rare disease cases has been shown to be around 40% for certain indications (Wright et al., 2023). For children with a rare disease, a diagnosis can provide both emotional and practical benefits for the parents, including relief from guilt, validation that the child has a condition, information about recurrence risk, information about prognosis, access to treatment, management and support groups, and additional social and/or educational support (Ashtiani et al., 2014; Griffin et al., 2017; Klitzman et al., 2024; Lewis et al., 2020; Peter et al., 2022). However, a timely diagnosis can be challenging, and in Europe, a recent survey conducted in 2022 found that the average time it took to receive a diagnosis was close to 5 years (Faye et al., 2024). Following on from the 100,000 Genomes Project in England, a pilot study to explore the use of WGS in a clinical setting, WGS for pediatric rare disease diagnosis is now available in England as a first‐line test for certain clinical indications (Robinson, 2020). Nevertheless, many patients will remain undiagnosed despite these advances, meaning that parents and families will continue to experience what has been coined the “diagnostic odyssey,” which refers to the often long, complex, and frustrating journey experienced in the search of a diagnosis (Lewis et al., 2010; Rosenthal et al., 2001).

Understanding the psychological impact of caring for a child with a rare and undiagnosed condition, as well as the parent and child characteristics that influence parents' psychological state, is vital to develop policies and practices that are family‐centered and improve family well‐being and long‐term outcomes for both parents and children. It can help to inform clinicians as to the types of psychological stressors families may be experiencing that they should look out for, as well as facilitate practices and interventions to aid coping. As part of a broader study to understand the clinical, psychosocial, and behavioral impact of receiving WGS results for pediatric patients (Best et al., 2021), we conducted an analysis of survey data to understand the psychological profiles of parents caring for a child with a rare and undiagnosed condition. We analyzed the data to address the following research questions: (1) What factors are associated with parental anxiety? (2) What factors are associated with parental health‐related quality‐of‐life and family functioning?

## 2 METHODS

### 2.1 Ethics statement

This study was reviewed by NHS Research Ethics Committee [London—Bloomsbury Research Ethics Committee, REC reference: 21/PR/0678].

### 2.2 Study design

This study forms part of a broader mixed‐methods study looking at the implementation of WGS in routine clinical care in the NHS in England (Lewis et al., 2022). This is a multi‐site quantitative survey study conducted at two time points: time 1 (T1) following consent—reported here in this manuscript, and time 2 (T2) following receipt of results (data collection is currently ongoing and will be reported at a later date). In this paper, we examined the relationship between parental anxiety and quality‐of‐life at T1.

### 2.3 Survey design measures and piloting

The design and piloting of the survey have been reported elsewhere (Patel et al., 2025), but in brief, these were informed by the research aims, a previous study conducted with participants in the 100,000 Genomes Project, and in collaboration with our study advisory and PPI (patient and public involvement) team. An initial list of measures and bespoke questions was reduced through consultation with the project advisory team and the PPI team.

### 2.4 Measures

The final version of the T1 survey included (in addition to items to measure knowledge, attitude, decisional conflict, and satisfaction which have been reported elsewhere (Patel et al., 2025)), the following measures (see Data S1 for the full survey).

*Parental anxiety* was assessed using the GAD‐7 anxiety measure (Spitzer et al., 2006). Responses are rated on a 4‐point scale. Response options range from 0 to 3 with total score ranges from 0 (minimum anxiety) to 21 (maximum anxiety). The severity of anxiety symptoms is categorized as 0–4: minimal anxiety, 5–9: mild anxiety, 10–14: moderate anxiety, 15–21: severe anxiety.

*Parental resilience* was assessed using the 6‐item Brief Resilience Scale (BRS) (Smith et al., 2008). Responses are rated on a 5‐point Likert scale. Negative items are reverse scored. The final BRS score ranges from 1 to 5, with 1.00–2.99 indicating low resilience, 3.00–4.30 indicating normal resilience, and 4.31–5.00 indicating high resilience.

*Tolerance for uncertainty* was assessed using a bespoke single question (“How well do you feel you deal with uncertainty in your life?”) with 5 response options ranging from “Not at all well” to “Very well.”

*Impact of the child's condition on parental HRQOL and family functioning* was assessed using the 37‐item PedsQL Family Impact Module (FIM) which measures parent self‐reported physical, emotional, social, and cognitive functioning, communication, and worry as well as daily activities and family relationships (Varni et al., 2004). Each item is rated on a 5‐point Likert scale. Items are reverse scored and linearly transformed to a 0–100 scale. Higher scores indicate better functioning or less impact on the family. As well as mean subscale scores and an overall score, Parent Health‐related Quality‐of‐Life (HRQOL) summary scores were calculated by averaging the scores of the Physical, Emotional, Social, and Cognitive subscales, and Family Functioning summary scores were calculated by averaging the scores of the Communication, Worry, Daily Activities, and Family Relationships subscales.

*Child health‐related quality‐of‐life* was assessed using the EQ‐5D‐Y‐3L, a 15‐item generic tool for patient‐reported outcomes, which is completed by a caregiver on behalf of their child. It comprises the following five dimensions: mobility, looking after myself, doing usual activities, having pain or discomfort, and feeling worried, sad, or unhappy (EuroQol Group, 1990). Each dimension has 3 levels: no problems, some problems, and a lot of problems. The measure has only been validated in children aged 4 and above. We therefore only asked parents of children aged 4 and over to complete this set of questions. To quantify health‐related quality‐of‐life using the EQ‐5D‐Y‐3L scores, responses were converted into a single index value using the United Kingdom EQ‐5D‐Y‐3L value set. This conversion applies preference weights based on the specific population. Index scores can range from −0.17 to 1, where negative values indicate a state worse than death and 1 indicates perfect health.

Participants were also asked to complete the EQ‐VAS, a component of the EQ‐5D‐Y‐3L, to describe how well they think the child's health is at the time of assessment. The EQ‐VAS is a vertical visual analogue scale, ranging from 0 to 100, where 0 indicates the worst imaginable health and 100 the best imaginable health. It adds a subjective layer to the EQ‐5D by allowing individuals to express their overall health perception in a simple, visual manner.

*Parent characteristics* measures included questions to assess gender, age, relationship to the patient, number of children, educational attainment, household income, ethnicity, and religious faith.

*Child characteristics* measures included age, how long they have been looking for a diagnosis, and whether they had previous genetic tests. Perceived severity of the rare condition was assessed with two items adapted from the Revised Illness Perceptions Questionnaire to assess the seriousness of the child's condition (Moss‐Morris et al., 2002) and the impact of the condition on the child's life, measured on a 5‐item Likert scale.

### 2.5 Participants and recruitment

This study involved parents of children aged 16 years or younger (at the time of recruitment) who had an undiagnosed rare disease. These parents attended appointments at one of seven collaborating NHS sites across England (in London, the south of England, and the north of England), where they discussed and/or provided consent for WGS. These appointments were held between May 2022 and August 2023.

Following their appointment, participants were sent survey packs by their respective NHS site within 4 weeks. Each pack included: (1) a paper survey (which also featured a QR code for online completion), (2) a participant information sheet, (3) a prepaid return envelope, and (4) an invitation letter from the NHS site. Returning the survey was considered as implied consent to participate in the study. At three of the seven sites, non‐responders were followed up either via telephone (at two sites) or through a second postal survey (at one site), though these follow‐ups were not conducted consistently across sites. As a token of appreciation, participants received a £10 gift voucher. At the end of the survey, participants had the option to provide their contact details to receive the second survey (T2), the gift voucher, and to potentially be invited for a follow‐up qualitative interview.

Additionally, data were collected from recruiting sites regarding the WGS panel requested by clinicians, enabling researchers to identify the clinical indication (labeled using an “R” number as specified in the National Genomic Test Directory [NGTD]). Information about the health professional who obtained consent from the parents was also gathered, categorized into three groups: (1) genetic counselors/genomics associates, (2) genetic consultants, or (3) non‐genetic consultants. A genomic associate provides administrative support for clinics, patients, and the clinical activities of genetic counselors and clinical geneticists.

### 2.6 Sample size

The primary outcome of the study was to compare decision regret at T2 between parents that received a diagnosis and those that did not. A minimum of 67 participants was therefore required in both groups to detect a medium effect size (0.5) with an 80% power level. As diagnostic rates using genomic testing are currently around 40% when trio‐based analysis is performed (Wright et al., 2023), a minimum of 168 participants was required. To account for drop‐out between the T1 and T2 survey, which was around 50% in previous research, we therefore aimed to recruit around 400 participants at T1 (Sanderson et al., 2022).

### 2.7 Data analysis

Statistical analyses were performed with the Statistical Package for the Social Science (SPSS) version 28. Due to small numbers within groups, some categorical variables were collapsed. Household income was categorized into low (£0–£30,000), medium (£30,001–£50,000), and high (£50,001 and over). Education was categorized as low (none to GCSE's [equivalent of finishing school at 16 years]), medium (A levels/BTEC [equivalent of finishing school at 18 years]), and high (Bachelors/Masters). Ethnicity was categorized as White and Non‐White. Religion was categorized as None, Christian, and Other.

We conducted bivariate analysis to explore relationships between variables. To assess relationships between continuous numeric variables, Kendall's tau‐b (*τb*) correlation coefficients were calculated. Analysis between categorical groups were conducted using Kruskal–Wallis tests, followed by pairwise Mann–Whitney *U* tests. Statistical significance was set at *p* < 0.05. Only variables that were significantly associated with the GAD‐7 and FIM scores in the bivariate analyses (*p* < 0.05) were entered into a multiple linear regression model to examine the relationship between multiple variables simultaneously and control for confounding factors. Variables that were not significant in bivariate analysis were excluded from the multivariable model to avoid overfitting. The performance of the model was assessed using adjusted *R* ^2^ and *F*‐statistics. Interaction terms were not tested due to sample size limitations. Regression results, including beta coefficients, standard errors, *p* values are presented.

## 3 RESULTS

### 3.1 Parent and child characteristics and psychological traits

In total, 1366 surveys were distributed across seven NHS Trusts in England with 383 returned. Of those, seven were excluded because of missing data (they only included demographic data and the remaining items had not been completed) and two were excluded because they were duplicated surveys where the same responder had completed the survey twice, leaving 374 surveys included in the final analysis (27% response rate).

The majority of survey respondents were female gender (89%), biological parent (91.7%), were White or White British ethnicity (81.5%), and had consented to WGS (94.7%) as well as the National Genomic Research Library (NGRL) (68.4%). Respondent characteristics are detailed in Table 1 along with details about parental decision‐making. Child characteristics are detailed in Table 2.

**TABLE 1:** Parent characteristics.

Characteristic	Response options	*N* (%)
Participant type	Biological parent	343 (91.7)
	Non‐biological parent	17 (4.5)
	Carer	5 (1.3)
	Other	3 (0.8)
	Missing	6 (1.6)
Gender	Female	333 (89)
	Male	38 (10.2)
	Another gender identity	1 (0.3)
	Missing	2 (0.5)
Age, years	Mean (SD), range	37.98 (7.73) 21–77
Number of children	Mean (SD), range	2.31 (1.23) 1–9
Education	No qualification	18 (4.8)
	GCSE or O level	63 (16.8)
	GCE, A level, or similar	37 (9.8)
	Vocational (BTEC/NVQ/Diploma)	60 (16)
	Bachelor's degree or equivalent	101 (27)
	Master's degree or equivalent	50 (13.4)
	PHD, MD, or equivalent	9 (2.4)
	Missing	36 (9.6)
Household income	Below £10,000	31 (8.2)
	£10,001–£30,000	95 (25.4)
	£30,001–£50,000	71 (19)
	£50,001–£70,000	61 (16.3)
	Over £70,001	61 (16.3)
	Missing	55 (14.7)
Ethnicity	Asian or Asian British	32 (8.6)
	Black or Black British	8 (2.1)
	Mixed	6 (1.6)
	White or White British	305 (81.5)
	Other ethnic group	11 (2.9)
	Missing	12 (3.2)
Religion	None	191 (51)
	Buddhist	1 (0.3)
	Christian/Catholic	142 (38)
	Hindu	1 (0.3)
	Jewish	4 (1.1)
	Muslim	27 (7.2)
	Sikh	3 (0.8)
	Other	4 (1.1)
	Missing	1 (0.3)
Did parent consent to WGS	Yes	354 (94.7)
	No	4 (1.1)
	Missing	16 (4.3)
Did parent consent to NGRL	Yes	256 (68.4)
	No	14 (3.7)
	Was not asked	16 (4.3)
	Don't know	85 (22.7)
	Missing	3 (0.8)
Type of health professional that consented parent	Genomic associate, genetic counselor or nurse	157 (42)
	Clinical geneticist or genetics registrar	146 (39)
	Non‐genetic consultant	14 (3.7)
	Information not available	33 (8.8)
	Not consented	5 (1.3)
	Missing	19 (5.1)
Mode of appointment	In person	251 (67.1)
	By telephone	76 (20.3)
	Virtual	20 (5.3)
	Missing	27 (7.2)
How well do you deal with uncertainty in your life?	Very well	25 (6.3)
	Well	133 (33.8)
	Neither well nor not well	116 (29.4)
	Not well	72 (18.3)
	Not at all well	25 (6.3)
	Missing	23 (5.8)
Parent resilience	Mean (SD), range	3.27 (SD = 0.75), 1–5
	Low resilience	101 (28.1)
	Normal resilience	230 (63.9)
	High resilience	29 (8.1)
	Missing	14 (3.7)
GAD‐7 anxiety	Mean (SD), range	5.31 (SD = 5.82), 1–5
	Minimal anxiety	199 (55.1)
	Mild anxiety	83 (23)
	Moderate anxiety	41 (8.1)
	Severe anxiety	38 (10.5)
	Missing	13 (3.3)

**TABLE 2:** Child characteristics.

Characteristic	Response options	*N* (%)
	Developmental disorders	199 (68.4)
Condition type	Neurology	55 (23.2)
	Ophthalmology	19 (9)
	Metabolic	11 (5.2)
	Musculoskeletal	11 (5.2)
	Ultra‐rare and atypical monogenic disorders	10 (4.8)
	Immunology	7 (3.3)
	Mitochondrial	5 (1.3)
	Endocrinology	5 (2.4)
	Cardiology	3 (1.4)
	Renal	3 (1.4)
	Hematology	1 (0.5)
Age, years	Mean (SD), range	6.2 (4.3), 0–17
Previous genetic tests?	Yes	194 (51.9)
	No	160 (42.8)
	Don't know	14 (3.7)
	Missing	6 (1.6)
Length of time looking for a diagnosis, years	Mean (SD), range	4.04 (2.48), 1–8
“My child's condition is serious”	Strongly agree	66 (17.6)
	Agree	120 (32.1)
	Neither agree nor disagree	126 (33.7)
	Disagree	47 (12.6)
	Strongly disagree	13 (3.5)
	Missing	2 (0.5)
“My child's condition has major consequences on their life”	Strongly agree	105 (28.1)
	Agree	127 (34)
	Neither agree nor disagree	90 (24.1)
	Disagree	34 (9.1)
	Strongly disagree	13 (3.5)
	Missing	5 (1.3)

### 3.2 Question 1: What factors are associated with parental anxiety?

Across all parents, the mean anxiety score as measured by the GAD‐7 was 5.31 out of 21 (*n* = 361, SD = 5.82) indicating that overall anxiety was low. Of those, 199 (55.1%) were categorized as having minimal anxiety, 83 (23.0%) had mild anxiety, 41 (11.4%) moderate anxiety, and 38 (10.5%) had severe anxiety.

To understand the relationship between parental anxiety and the demographic and psychological factors that we measured, we first conducted a series of bivariate analyses in which anxiety score was the dependent variable. We found that anxiety was associated with parents' age, with younger parents experiencing higher levels of anxiety than older parents (*τb* = −0.08, *p* = 0.034). Anxiety was also associated with gender, with females being more anxious than men (mean = 5.68 vs. 2.39 respectively; *U* = −4059.5; *Z* = −3.429, *p* < 0.001). Anxiety was also associated with education (H (2) = 8.73, *p* = 0.013), with post‐hoc analysis indicating that parents with the lowest educational attainment had higher anxiety than those in the medium attainment group (*p* = 0.037) and similarly those in the medium educational attainment group had higher anxiety than those in the highest group (*p* = 0.024) suggesting that the more educated the parent is, the lower their anxiety is. Higher parent resilience scores and better tolerance for uncertainty were also significantly associated with lower anxiety (*τb* = −0.345, *p* < 0.001 and *τb* = −0.32, *p* < 0.001, respectively). Anxiety was not associated with any other parent demographic characteristics (ethnicity, religion, household income), nor the mode of appointment nor type of healthcare professional conducting the consent conversation.

Regarding child characteristics, parental anxiety was positively associated with both the seriousness of the child's condition (*τb* = 0.120, *p* = 0.004) and the consequences of the condition on the child's daily life (*τb* = −0.314, *p* < 0.001). The more serious the child's condition and the greater the consequence, the more anxious the parents were. We also looked at whether anxiety was associated with the child health‐related quality‐of‐life, as measured by the EQ‐5D‐Y‐3L (for children aged 4 and over only). The average score was 0.57 (*n* = 178, SD = 0.27, range = −0.17–0.97; Table 3). Participants' total scores were significantly associated with lower anxiety, indicating that the better the child's health‐related quality‐of‐life, the lower parental anxiety (*τb* = −0.175, *p* = 0.005). The average score on the EQ‐VAS (which measures how well a parent *thinks* the child's health is at the time of assessment) was 68.01 (*n* = 229, SD = 21.19, range = 15–100). As with the EQ‐5D‐Y‐3L, higher EQ‐VAS scores were significantly associated with lower anxiety scores, indicating that the better the parents' perception of their child's health, the lower their anxiety (*τb* = −0.115, *p* = 0.020). Anxiety was not associated with the child's age, whether they had a previous genetic test before, or the length of time spent looking for a diagnosis.

**TABLE 3:** Child health‐related quality‐of‐life as measured on the EQ‐5D‐Y‐3L.

EQ‐5D‐Y‐3L subscale	Answer options	*N* (%)
EQ‐5D‐Y‐3L: Mobility^a^	No problems	111 (47.4)
	Some problems	88 (37.6)
	A lot of problems	31 (13.2)
	Missing	4 (1.7)
EQ‐5D‐Y‐3L: Looking after him/herself^a^	No problems	60 (25.6)
	Some problems	89 (38.0)
	A lot of problems	80 (34.2)
	Missing	5 (2.1)
EQ‐5D‐Y‐3L: Doing usual activities^a^	No problems	47 (20.1)
	Some problems	104 (44.4)
	A lot of problems	78 (33.3)
	Missing	5 (2.1)
EQ‐5D‐Y‐3L: Having pain or discomfort^a^	No pain or discomfort	110 (47.0)
	Some pain or discomfort	98 (41.9)
	A lot of pain or discomfort	20 (8.5)
	Missing	6 (2.6)
EQ‐5D‐Y‐3L: Feeling worried, sad, or unhappy^a^	Not worried, sad, or unhappy	54 (23.1)
	A bit worried, sad, or unhappy	70 (29.9)
	Very worried, sad, or unhappy	24 (10.3)
	Missing	86 (36.8)

^a^ The EQ‐5D‐Y‐3L scale is only validated for children aged 4 years and over (*n* = 234 in our sample).

For the multivariable analysis, the variables that were significantly associated with parental anxiety in the bivariate analyses were retained in the final model (Table 4). The analysis revealed that higher anxiety scores remained significantly associated with parental age, higher educational attainment, resilience, and tolerance for uncertainty. The model was statistically significant, *F*(9, 132) = 7.76, *p* < 0.001, and accounted for approximately 30.1% of the variance in anxiety scores (adjusted *R* ^2^ = 0.301).

**TABLE 4:** Anxiety multivariable regression model.

Variable	*B* (Unstandardized Coef.)	SE	Beta (Standardized Coef.)	*t*	*p*‐value	Tolerance	VIF
Parents' age	−0.141	0.056	−0.179	−2.62	**0.010**	0.944	1.059
Education	2.550	0.463	−0.086	2.95	**0.004**	0.936	1.069
Resilience	−1.363	0.786	−0.123	−1.99	**0.049**	0.599	1.670
Tolerance for uncertainty	−1.104	0.423	−0.295	−2.34	**0.021**	0.642	1.557
EQ‐5D‐Y‐3L	−2.721	1.72	−0.138	−1.582	0.116	0.652	1.535
EQ‐VAS	−0.015	0.022	−0.057	−0.697	0.487	0.753	1.328
Parents' gender	−1.786	1.171	−0.11	−1.526	0.129	0.959	1.043
“My child's condition is serious”	0.058	0.481	0.011	0.121	0.904	0.557	1.794
“My child's condition has major consequences”	0.852	0.496	0.17	1.718	0.880	0.505	1.978
Constant	18.686	3.965	–	4.713	<0.001	–	–

Significant values at *p* < 0.05 are in bold.

### 3.3 Question 2: What factors impact parental quality‐of‐life and family functioning?

#### 3.3.1 PedsQL Family Impact Module (FIM) total score

**FIGURE 1:**
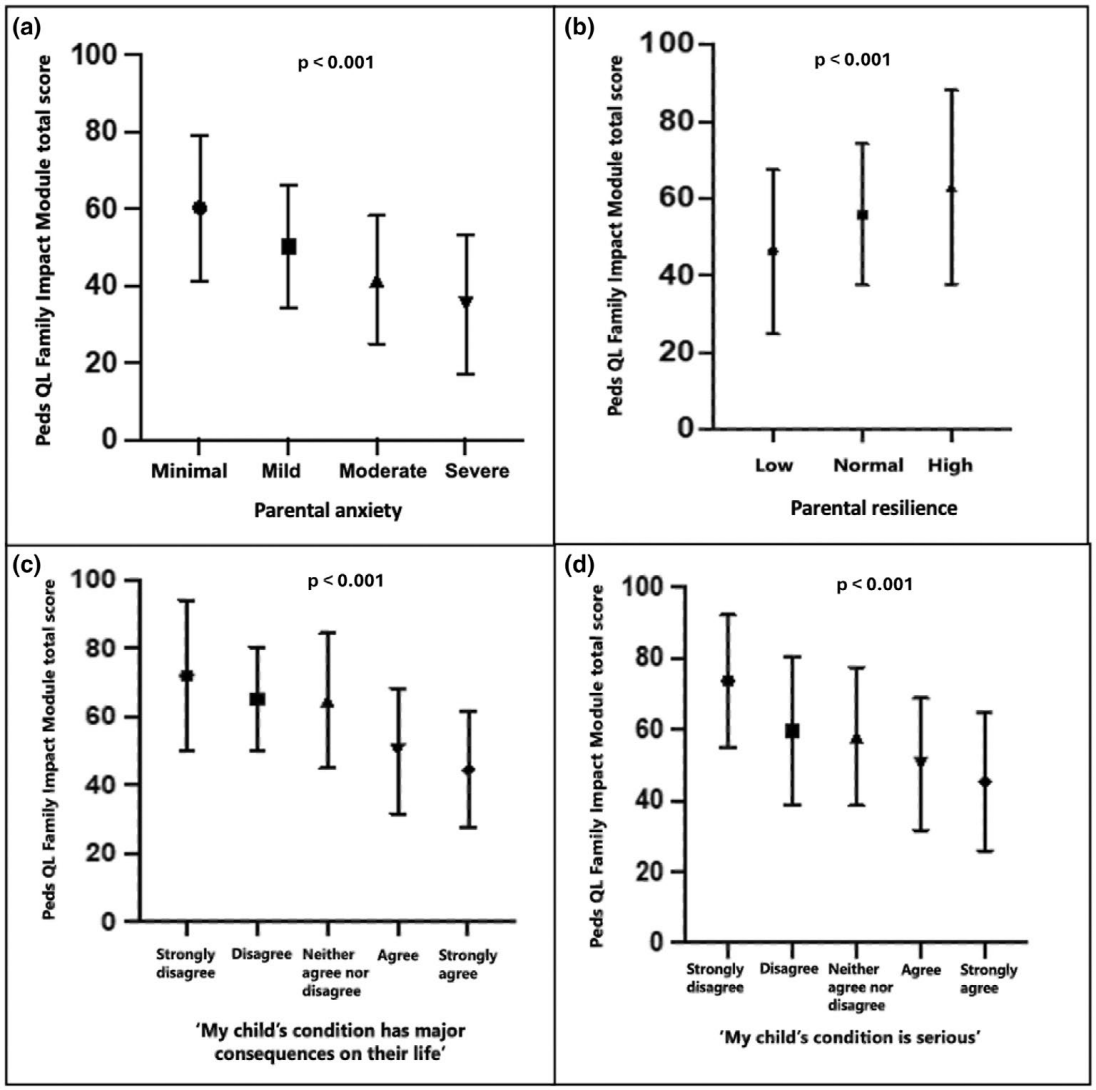
Impact of parental and child characteristics on parent quality‐of‐life and family functioning.

**FIGURE 2:**
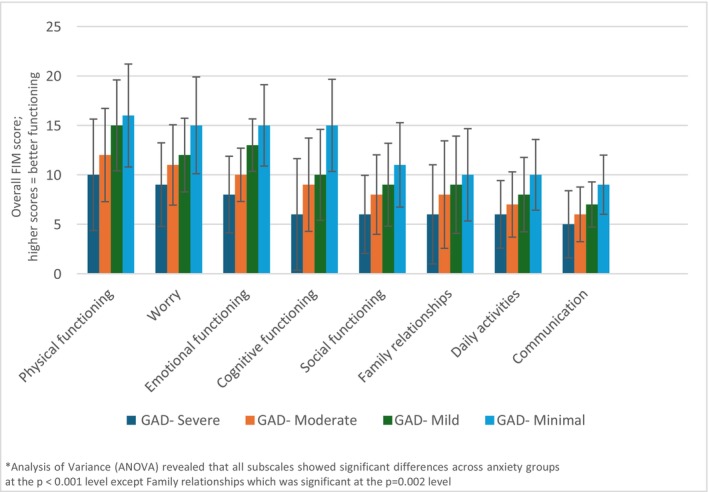
PedsQL family impact subscales compared with anxiety.

The mean FIM total score was 53.68 (*n* = 330, SD = 20.45, Range 0–100), with higher scores indicating better parental quality‐of‐life and family functioning (Table 5). To understand the relationship between FIM and the demographic and psychological factors that we measured, we first conducted a series of bivariate analyses in which anxiety score was the dependent variable. We found that total FIM score was negatively associated with anxiety, with parents with higher anxiety experiencing lower FIM scores, indicative of worse quality‐of‐life and family functioning (*τb* = −0.377, *p* < 0.001; Figure 1a), including across all the subscales (Figure 2). Lower FIM scores were also significantly associated with lower parental resilience (Figure 1b) and lower tolerance for uncertainty (*τb* = 0.197, p < 0.001 and *τb* = 0.163, *p* < 0.001, respectively; Figure 1). Total FIM scores were not significantly associated with parents' age, gender, ethnicity, religion, income, or educational attainment.

**TABLE 5:** Family Impact Module (FIM) scale including summary scores and subscales.

	Mean (possible scores)	SD
FIM total score (*N* = 330)	53.68 (0–100)	20.45
Health‐related quality‐of‐life summary score (*N* = 352)	54.53 (0–100)	21.34
Physical functioning (*N* = 368)	52.45 (0–100)	23.18
Emotional functioning (*N* = 369)	52.59 (0–100)	22.91
Social functioning (*N* = 371)	54.39 (0–100)	27.56
Cognitive functioning (*N* = 372)	59.25 (0–100)	26.24
Family functioning summary score (*N* = 330)	56.22 (0–100)	24.18
Daily activity (*N* = 372)	43.68 (0–100)	30.34
Worry (*N* = 372)	47.29 (0–100)	24.65
Communication (*N* = 370)	51.31 (0–100)	25.41
Family relationship (*N* = 373)	63.59 (0–100)	25.01

When looking at child characteristics, total FIM scores were significantly associated with the seriousness of the child's condition and consequences on the child's daily life, with parents of children whose condition was judged to be more serious and have more consequences having lower FIM scores (*τb* = −0.255, *p* < 0.001 and *τb* = −0.314, *p* < 0.001, respectively; Figure 1c,d). Parents whose children were younger (*τb* = −0.081, *p* = 0.028), had previous genetic testing (*U* = 9615, *z* = −3.424, *p* < 0.001), had spent longer looking for a diagnosis (*τb* = −0.152, *p* < 0.001), and whose child scored lower on the EQ‐5D‐Y‐3L and EQ‐VAS measures also had lower FIM scores (*τb* = 0.400, *p* < 0.001 and *τb* = 0.291, *p* < 0.001, respectively).

For the multivariable analysis, the variables that were significantly associated with the FIM in the bivariate analyses were retained in the final model. After multivariable analysis, the EQ‐5D‐Y‐3L and EQ‐VAS, which measure child HRQOL, remained significantly associated, as did anxiety. In other words, parents of children with lower HRQOL also had worse HRQOL and family functioning, as well as higher anxiety (Table 6). The model was statistically significant, *F*(10, 125) = 17.48, *p* < 0.001, and explained 55.0% of the variance in family impact scores (*adjusted R* ^2^ = 0.550).

**TABLE 6:** Family Impact Module multivariable regression model.

Variable	*B* (Unstandardized Coef.)	SE	Beta (Standardized Coef.)	*t*	*p*‐value	Tolerance	VIF
Parental anxiety	−1.095	0.29	−0.269	−3.856	**<0.001**	0.665	1.504
EQ‐5D‐Y‐3L	24.784	6.077	0.311	4.172	**<0.001**	0.626	1.597
EQ‐VAS	0.175	0.079	0.16	2.152	**0.025**	0.652	1.535
Parental resilience	3.222	2.449	0.116	1.316	0.191	0.429	2.333
Tolerance for uncertainty	1.966	1.749	0.095	1.124	0.263	0.47	2.126
Previous genetic testing	2.715	2.448	0.067	1.109	0.269	0.901	1.11
Length of time spent looking for a diagnosis	−0.75	0.75	−0.083	−1	0.319	0.489	2.043
Child age	0.026	0.474	0.005	0.055	0.956	0.484	2.065
“My child's condition is serious”	−2.29	1.72	−0.104	−1.332	0.185	0.543	1.842
“My child's condition has major consequences on their life”	−2.36	1.804	−0.11	−1.308	0.193	0.47	2.127
(Constant)	31.319	12.173	–	2.573	0.011	–	–

Significant values at *p* < 0.05 are in bold.

#### 3.3.2 Parent health‐related quality‐of‐life and family functioning summary scores

We also looked specifically at the two summary scores, parent HRQOL and family functioning. The mean HRQOL summary score was 54.53 (*n* = 352, SD = 21.34, Range 0–100). The lowest mean score, indicating lowest HRQOL, was on the physical functioning subscale (52.53) with the next lowest score on the emotional functioning subscale (52.45). Bivariate analysis indicated that higher anxiety (*τb* = −0.402, *p* < 0.001), lower resilience (*τb* = 0.221, *p* < 0.001), and less tolerance for uncertainty (*τb* = 0.2, *p* < 0.001) were all associated with lower parental HRQOL. The mean family functioning summary score was 56.22 (*n* = 330, SD = 24.18, Range 0–100). The lowest mean score, indicating lowest family functioning, was on the daily activity subscale (43.68) with the next lowest score on the worry subscale (47.29). Family functioning scores were significantly worse for parents of children with developmental disorders compared to those whose children had other condition types (mean = 132.66 vs. 157.89 respectively; *U* = 6883; *Z* = ‐2.414, *p* = 0.016). Bivariate analysis also indicated that higher anxiety (*τb* = −0.276, *p* < 0.001), lower resilience (*τb* = 0.12, *p* = 0.001), and less tolerance for uncertainty (*τb* = 0.08, *p* = 0.047) were all associated with poorer family functioning.

## 4 DISCUSSION

To our knowledge, this is the largest study to date looking at parental anxiety, HRQOL, and family functioning, when caring for a child with a rare and undiagnosed condition. In answer to our first question, “what factors are associated with parental anxiety?,” our findings indicate that around one fifth of parents were experiencing moderate to severe anxiety, and that anxiety was higher amongst younger parents and those with lower educational attainment, as well as amongst parents who were less resilient and had lower tolerance for uncertainty. In answer to our second question, “what factors are associated with parental HRQOL and family functioning?,” we found that parental HRQOL and family functioning were lower amongst parents who were more anxious and whose child had poorer HRQOL, with emotional and physical functioning the two HRQOL aspects most impacted by their child's condition. Our findings highlight the interconnectedness between the psychological constructs anxiety, resilience, tolerance for uncertainty, and parental QOL.

Unsurprisingly, when comparing our findings to population norms, we found that parents of children with undiagnosed rare conditions are more anxious compared with the general parent population, as measured on the GAD‐7. In the US, parents of school‐age children (aged between 5 and 12 years) from the general population had a mean score of 3.4 compared with 5.31 in our sample (Sequeira et al., 2021). Our findings are more in line with research conducted with parents of children with chronic and/or rare conditions (Graziano et al., 2023), and in particular, children with undiagnosed conditions, where the mean score for parents was 4.85 (mean = 4.89 for females) in a study conducted in the USA (McConkie‐Rosell et al., 2018). In our study, we also found that around one fifth of parents were experiencing moderate to severe anxiety. These findings cause concern, not only because of the significant associated impairments anxiety has on parental social and occupational functioning (Mendlowicz & Stein, 2000) but also because there is an established body of literature linking parental anxiety to psychiatric outcomes in children (Halligan et al., 2007; Nomura et al., 2002). This also extends to children affected by rare conditions. For example, in a study looking at patients with cystic fibrosis and their parent caregivers, Quittner et al. (2014) found that parental depression or anxiety doubled the risk that the affected child reported elevated psychological distress. In a study looking at the experience of parents of children with special needs during the COVID‐19 pandemic, parental symptoms of generalized anxiety and depression were found to be associated with adverse mental health symptoms and health‐related behaviors in their children (Gruszka et al., 2023). Around half of parent carers have never been asked about their emotional well‐being by health professionals, suggesting a lack of awareness of the emotional toll of caring for a child with a rare condition (Spencer‐Tansley et al., 2022). Healthcare providers should, at a minimum, be able to identify parents who may be experiencing elevated levels of anxiety, provide emotional and psychological support including ways of managing anxiety, and/or refer them to psychological support services. This may include cognitive‐behavioral therapy (CBT) which has been found to improve depression and anxiety in parents of children after the end of treatment for childhood cancer (Ljungman et al., 2018) as well as mindfulness‐based stress reduction (MBSR) to foster emotional regulation, which has been found to reduce stress and increase life satisfaction in parents of young children with developmental delays (Neece, 2014).

Resilience and tolerance for uncertainty were identified as key factors that impact anxiety. Resilience is a construct that is concerned with exposure to adversity and the positive outcomes of that adversity (Luthar & Cicchetti, 2000). It is perceived not as a fixed attribute, but as a dynamic process that can change over time, dependent on an individual's specific context and their interaction with their environment (Luthar & Cicchetti, 2000). As such, strategies to enhance resilience and cope with uncertainty could reduce parental anxiety. This could include incorporating approaches such as acceptance and commitment therapy (ACT) or CBT into genetic counseling training so that interventions to help parents change their relationship with their thoughts can be provided by those already involved in the testing pathway (Davies et al., 2024).

Drawing on Bronfenbrenner's ecological systems theory (Bronfenbrenner & Morris, 2006), healthcare professionals and the genomic medicine service more broadly have an important role to play. This includes: timely and accessible information giving, as well as discussing parents' preferences for being told uncertain information prior to testing (Harding et al., 2020; the microsystem); linking parents with rare disease communities through patient groups as well as signposting to psychological support where available (Hammond et al., 2022; the mesosystem); ensuring timely access to genomic testing to reduce the diagnostic odyssey, which has been identified as an important aspect of improving parents' experience, mental well‐being, and quality‐of‐life (Jones et al., 2024; the exosystem); and improving genomic literacy so that societal, cultural, or religious attitudes that perpetuate stigma, shame, or guilt around rare diseases are reduced (Boardman et al., 2020; Chediak et al., 2022) (the macrosystem). In fact, most parents in this study were experiencing minimal anxiety, which might tentatively suggest that many parents of children with undiagnosed rare conditions have developed strong resilience and coping skills (although we recognize parents with lower resilience might be less likely to have responded to the survey). Qualitative interviews to explore their experiences and methods of coping could improve our understanding of the internal and external factors that contribute to positive adaptation, which could in turn guide the development of psychosocial resources to support those that are experiencing elevated anxiety.

Anxiety was significantly associated with FIM scores, which is not surprising given that emotional functioning was the second lowest scoring subscale in the HRQOL summary score and worry was the second lower scoring subscale in the family functioning summary score. Parents in our sample had poorer FIM scores than parent population norms, which have mean scores of around 70–75 on the FIM (Medrano et al., 2013; Panepinto et al., 2009), and are more comparable to studies of parents of children with complex chronic health conditions (mean = 51.9; Mann et al., 2019), severe cerebral palsy/birth defects (mean = 62.49; Varni et al., 2004), and developmental delay (mean = 60.6; Hsieh et al., 2013). This could reflect that the majority of parents in this survey have a child with a developmental disorder. Beyond family functioning, the relationship between caring for a child with developmental delay and high parental stress levels, poorer mental health outcomes, and low QoL has been described in several studies (Dikow et al., 2019; Lee, 2013; Scherer et al., 2019), with disability severity associated with higher rates (Dikow et al., 2019; Scherer et al., 2019). Indeed, in this study, approximately half of parents agreed or strongly agreed that their child's symptoms were serious, which was strongly associated with parental anxiety and FIM scores. Another explanation could include that the relatively low mean score among our sample compared with other studies of parent carers is linked to the fact that the parents in our study are living in a state of uncertainty due to the fact that parents in this sample did not yet have a diagnosis. Developmental disorders are often variable and associated with an uncertain prognosis, and this uncertainty likely results in lower adaptation to their child's condition. This hypothesis is supported by results from a study of mothers of children with developmental and intellectual disabilities, where mothers' quality‐of‐life following the receipt of a diagnosis increased, providing further insight into the impact of uncertainty on maternal well‐being (Lingen et al., 2016).

Moreover, parents of children with undiagnosed conditions face challenges likely to be more pronounced due to the fact they do not have a diagnosis. This includes having to push or fight to access services, including educational support, therapeutic services, and specialized equipment, in part due to the lack of a “label” which makes form filling and assessment more complicated, leaving parents feeling emotionally drained (Aldiss et al., 2021; Lewis et al., 2010; Yanes et al., 2017). At the same time, research has shown that these parents are experiencing isolation, loss of hope, frustration at the lack of knowledge available about their child's condition alongside emotional worries, including fear of what the future holds (Aldiss et al., 2021; Gurasashvili et al., 2024; Krabbenborg et al., 2016; Yanes et al., 2017), factors likely contributing to poor quality‐of‐life, and family functioning experienced by parents in this study.

The finding that physical functioning was the poorest scoring subscale on the HRQOL summary score is interesting. We hypothesize this may be because intense parenting leaves parents feeling physically exhausted with little time to engage in self‐care including physical exercise (Aldiss et al., 2021). In a qualitative study looking at the mental health impact of caring for a child with an undiagnosed and rare condition conducted as part of this broader mixed‐methods study, parents were found to assume the role of the “hero” archetype and pushed themselves to their own mental and physical limits, resulting in parents denying their own needs around care and comfort (Hoffmann et al. manuscript under review). As one parent from that study said; “I wake up feeling tired, I go to sleep feeling tired.” As well as enquiring about parents' emotional well‐being, health professionals should ask parents questions to ascertain whether they are looking after their own physical health including staying active and getting enough sleep. If necessary, they should signpost parents to support such as respite care for carers or care coordinators, although research has found these to be poorly coordinated in the UK (Walton et al., 2023).

### 4.1 Implications for genetic counselors

In light of our findings, we have identified a number of proactive approaches genetic counselors could use to improve parental anxiety and quality‐of‐life:

1. Proactively assess parental anxiety, as well as factors that may exacerbate anxiety such as low resilience and intolerance for uncertainty, and recognize where referral for onward psychological support is required;
2. Prepare parents ahead of testing for possible uncertain outcomes and explore their perception of that uncertainty. Set realistic expectations around timelines for return of results as well as diagnostic yield. Describe how even with a diagnosis, there may still be uncertainty associated with their child's condition;
3. Support resilience building to combat anxiety. This may be through evidence‐based interventions, for example, CBT and mindfulness programs, as well as encouraging self‐care practices and realistic boundary settings in caregiving roles. Build these skills into the training program of genetic counselors;
4. Act as advocates or care coordinators where possible, including helping parents navigate systems, access supports, and understand how to document and communicate their child's needs;
5. Be attentive to the burden of the diagnostic odyssey and undiagnosed status. Validate the emotional impact of not having a diagnosis, including frustration, loss of hope, and system‐navigation fatigue. Provide practical support, for example, letters and/or resources where possible;
6. Facilitate connection to peer and emotional support through signposting parents to rare disease support groups or SWAN UK (https://geneticalliance.org.uk/support‐and‐information/swan‐uk‐syndromes‐without‐a‐name/) for families with an undiagnosed genetic condition. In the UK, the support group Rare Minds has a webpage dedicated to resources and information to support well‐being https://www.rareminds.org/wellbeing‐hub/. Recognize this as not just emotional support but also a strategy for improving QOL and reducing uncertainty through shared experience.

### 4.2 Strengths and limitations

Our study is strengthened by the large sample size and the use of validated measure, allowing us to compare our results to those of other published studies. Nevertheless, our response rate was low, increasing the potential for self‐reporting bias and thus a lack of representativeness. In addition, our sample was primarily of white ethnicity, well‐educated, and relatively affluent, and therefore does not necessarily reflect the psychological experiences of more marginalized, historically underserved communities. Finally, we were unable to collect the demographic data and condition type of non‐responders, so we do not know how representative our sample was of parents undergoing WGS more broadly. Nevertheless, our findings yield important insights into the psychological well‐being of parents caring for a child with a rare condition.

## 5 CONCLUSION

Our results shine a light on the mental health burden and quality‐of‐life impact of caring for a child with a rare, undiagnosed condition. Health professionals should be alert to and proactively inquire about parents' emotional state so they can identify those who might be experiencing poor psychological functioning, high levels of anxiety, and/or poor health‐related quality‐of‐life. Providing families with emotional support as well as access to services and resources to improve well‐being and cope with the challenges of caregiving is key. Further research to understand whether receipt of a genetic diagnosis has a positive impact on these psychological constructs is important. In our T2 survey, we will reevaluate this cohort and compare psychosocial health and functioning in those parents for whom a diagnosis is made with those who are still undiagnosed following WGS.

## AUTHOR CONTRIBUTIONS

RP conducted data analysis and interpretation and wrote the first draft of the manuscript; BF helped design the survey, collected data, and conducted data analysis and interpretation; SCS supported data analysis and interpretation; HE supported data analysis and interpretation; CL conceived of the study, designed the survey, supported data analysis and interpretation, and wrote the first draft of the manuscript. All authors reviewed and revised the manuscript.

## FUNDING INFORMATION

This research is funded through an NIHR Advanced Fellowship Grant (NIHR300099), awarded to Celine Lewis. The views expressed are those of the author(s) and not necessarily those of the NIHR or the Department of Health and Social Care.

## CONFLICT OF INTEREST STATEMENT

Ria Patel declares she has no conflict of interest. Bettina Friedrich declares she has no conflict of interest. Saskia Sanderson declares she has no conflict of interest. Holly Ellard declares she has no conflict of interest. Celine Lewis declares she has no conflict of interest.

## ETHICS STATEMENT

Human studies and informed consent: This study was reviewed and gained approval by the NHS Research Ethics Committee [London—Bloomsbury Research Ethics Committee, REC reference: 21/PR/0678]. Potential participants were sent a participant information sheet about the study. Returning a completed survey was taken as implied consent to participate.

Animal studies: No non‐human animal studies were carried out by the authors for this article.

## Supporting information


Data S1.

## Data Availability

Deidentified participant data are available from the corresponding author, Dr. Celine Lewis (celine.lewis@ucl.ac.uk), upon reasonable request and on the agreement it will not be made publicly available.
